# Sulfonated Polysulfone/TiO_2_(B) Nanowires Composite Membranes as Polymer Electrolytes in Fuel Cells

**DOI:** 10.3390/polym13122030

**Published:** 2021-06-21

**Authors:** Maria Jose Martinez-Morlanes, Carmen de la Torre-Gamarra, María Teresa Pérez-Prior, Sara Lara-Benito, Carmen del Rio, Alejandro Várez, Belen Levenfeld

**Affiliations:** 1 Department of Materials Science and Engineering and Chemical Engineering, Universidad Carlos III de Madrid, IAAB, Avda, Universidad, 30, E-28911 Leganés, Spain; mjm230181@gmail.com (M.J.M.-M.); catorreg@ing.uc3m.es (C.d.l.T.-G.); maperezp@ing.uc3m.es (M.T.P.-P.); 100304213@alumnos.uc3m.es (S.L.-B.); bll@ing.uc3m.es (B.L.); 2 Instituto de Ciencia y Tecnología de Polímeros (ICTP-CSIC), Calle Juan de la Cierva, 3, E-28006 Madrid, Spain; cdelrio@ictp.csic.es

**Keywords:** sulfonated polysulfone, composite membrane, TiO_2_(B) nanowires, proton-exchange membrane fuel cell

## Abstract

New proton conducting membranes based on sulfonated polysulfone (sPSU) reinforced with TiO_2_(B) nanowires (1, 2, 5 and 10 wt.%) were synthesized and characterized. TiO_2_(B) nanowires were synthesized by means of a hydrothermal method by mixing TiO_2_ precursor in aqueous solution of NaOH as solvent. The presence of the TiO_2_(B) nanowires into the polymer were confirmed by means of Field Emission Scanning Electron Microscopy, Fourier transform infrared and X-ray diffraction. The thermal study showed an increase of almost 20 °C in the maximum temperature of sPSU backbone decomposition due to the presence of 10 wt.% TiO_2_(B) nanowires. Water uptake also is improved with the presence of hydrophilic TiO_2_(B) nanowires. Proton conductivity of sPSU with 10 wt.% TiO_2_(B) nanowires was 21 mS cm^−1^ (at 85 °C and 100% RH). Under these experimental conditions the power density was 350 mW cm^−2^ similar to the value obtained for Nafion 117. Considering all these obtained results, the composite membrane doped with 10 wt.% TiO_2_(B) nanowires is a promising candidate as proton exchange electrolyte in fuel cells (PEMFCs), especially those operating at high temperatures.

## 1. Introduction

Global warming produced by the combustion of fossil fuels together with the continuous increase in energy consumption is currently leading to a search for new, clean, and efficient ways of using energy. Thus, green energy technologies such as solar cells, wind and hydro power, fuel cells or batteries, have recently attracted the attention of many researchers. All of these technologies are focused to overcome the problem of environmental contamination as well as the energy production and storage. In particular, proton exchange membrane fuel cells (PEMFCs) are a promising and competitive energy source for portable, vehicular and stationary applications because they can produce high power densities working at low temperature conditions, low emissions and fast charge responses [1,2,3].

Among the different PEMFCs components, the proton exchange membrane is the key part of the electrochemical system, which aim is to transfer protons from the anode to the cathode avoiding the fuel gas cross-leak between the electrodes. Nowadays, Nafion is the most widely used polymer electrolyte in PEMFCs due to its good chemical stability and high ionic conductivity. However, its low electrochemical and mechanical stability at high temperatures along with its high cost, are the main drawbacks of this material [4,5]. As a consequence, several alternative membranes based on thermostable aromatic polymers with non-perfluorinated backbone, have recently being developed. Specifically, polymers such as poly(arylene ether sulfone)s [6], poly(ether ether ketone)s [7], poly(sulfide sulfone)s [8], polyimides [9], polyphosphazenes [10] and polybenzimidazoles [11] have been investigated. Between these materials, polysulfone (PSU) is the most promising due to its low cost, easy processability and commercial availability [12]. This aromatic polymer can be sulfonated by treatments with different sulfonating agents, such as chlorosulfonic acid or trimethylsilyl chlorosulfonate, to produce a proton conducting material [13,14,15].

On the other hand, many studies have been carried out with the aim to improve the conductivity and thermal stability by dispersing inorganic nanoparticles. These fillers, generally with a hydrophilic nature, are intended to achieve both good stability at high temperatures and an increase of the absorption ability of water. Furthermore reduce the fuel-oxygen cross-over and improve mechanical properties. In this regard, the most studied inorganic fillers for polymer proton exchange membranes are SiO_2_ [16,17,18], zeolites [19], zirconium hydrogen phosphate (ZrP) [20], and TiO_2_ [21,22,23,24,25]. Among all these options, TiO_2_ is a good choice as an inorganic filler because it leads to a good chemical stability and good hydration to the polymer due to its high capability of absorbing water [26]. So, the incorporation of this inorganic solid into nafion membranes improves the mechanical and thermal properties of the polymer electrolyte [27]. Although there are several chemical and physical methods to prepare TiO_2_ with various morphologies, hydrothermal method is the most used due to its simplicity [28,29]. Among the different titanium dioxide polymorphs, TiO_2_(B) is composed by edge and corner sharing TiO_6_ with a slightly lower density compared with rutile, anatase or brookite polymorphs [30]. This fact makes TiO_2_(B) an interesting material for a wide variety of applications such as catalytic supports, solar energy conversion or intercalation electrodes for lithium batteries [31].

Considering all these aspects, the purpose of this experimental work is to prepare novel sulfonated polysulfone membranes reinforced with TiO_2_(B) nanowires prepared by casting procedure. The selection of sulfonated polysulfone is due, as indicated above, to its lower cost and good performances at high temperature and the choice of TiO_2_(B) nanowires is based on its hidrophilic character and suitable hydration during fuel cell operation. Although these nanowires have been used as reinforcement in nafion membranes, up to now and as far as we know, no membranes loaded with this hydrophilic filler have been reported in sulfonated polysulfone for fuel cell applications. Thus, in the present work, TiO_2_(B) nanowires have been used as inorganic filler to prepare by casting procedure, novel hybrid sulfonated polysulfone/TiO_2_(B) nanowire membranes. These nanowires were prepared by hydrothermal synthesis. Finally, the properties of the obtained membranes were examined in order to evaluate their possible application as proton exchange membranes.

## 2. Materials and Methods

### 2.1. Materials and Reagents

Polysulfone Udel (Mn = 22,000 g mol^−1^, PSU) was used as the polymer for membrane preparation. Trimethylsilyl chlorosulfonate (ClSO_3_Si(CH_3_)_3_, TMSCS, 99%), N,N-dimethylacetamide (DMAc) and 1,2-dichlorethane (DCE), were used as solvents and they were supplied by Sigma-Aldrich (Munich, Germany). For the TiO_2_(B) nanowires synthesis, titanium (IV) oxide nanoparticles (P25, Sigma-Aldrich (Munich, Germany), 99.5%, 21 nm particle size), NaOH (Labkem (Barcelona, Spain)) and HCl (Panreac (Barcelona, Spain), 37%) were employed. All the reagents and solvents were used as received with no previous treatments.

### 2.2. Preparation of TiO_2_(B) Nanowires

TiO_2_(B) nanowires were prepared by hydrothermal synthesis based on a previous work reported by Yoshida et al. [32]. Thus, 1 g of TiO_2_ nanoparticles powder (commercial) was added to 25 mL of and highly concentrated NaOH aqueous solution ([NaOH] = 10 M). The suspension was stirred for 24 h at room temperature and then transferred to a 40 mL volume Teflon-lined autoclave, which was subsequently heated at 150 °C during 120 h. Then, the mixed was naturally cooled down to room temperature and a white paste was obtained, which was acid-washed with HCl 0.1 M for 24 h and subsequently centrifuged. This step was repeated for different times until all NaOH was completely removed. Afterwards, the obtained white precipitate was washed with distiller water for eliminating HCl excess, centrifuged and vacuum filtered. The resulting white product was dried at 70 °C for 15 h to obtain hydrogen titanate (H_2_Ti_3_O_7_) nanowires. Finally, in order to prepare different TiO_2_ polymorphs, the as-synthetized titanate nanowires were heated at different temperatures between 100 °C and 700 °C for 2 h in an air atmosphere. The oven was pre-heated at the desired temperature before introducing the alumina crucible containing the titanium oxide powder.

### 2.3. Polysulfone Sulfonation

Sulfonated polysulfone (sPSU) was prepared by an electrophilic aromatic substitution according to the reference [33] and used in our laboratory as well [14]. At first, polysulfone was dissolved in DCE and the resulted solution was purged with N_2_ for 1 h. Subsequently, a solution with the TMSCS in DCE was added drop by drop and the resulted mixture was magnetic stirred for 24 h at room temperature. When the reaction was completed, the product was treated with ethanol, obtaining a precipitated sPSU, which was subsequently filtered and washed with distilled water until reaching pH = 7. Finally, the sPSU was dried at room temperature to constant weight.

### 2.4. Preparation of sPSU/TiO_2_(B) Membranes

All membranes of the study were prepared by casting from polymer solutions according to our previous works [14,16]. First, a solution with a concentration of 5 wt.% of sPSU in DMAc was stirred at room temperature and after 2 h it was filtered. For the preparation of composites, different amounts of TiO_2_(B) nanowires (1, 2, 5 and 10 wt.%) were added to the polymer solution and the resulting solutions were mechanically stirred at 60 °C during 1 h. After that, the solutions were sonicated at room temperature during 1 h and then mechanically stirred for an additional hour. Finally, the solutions were casted in a Petri glass dish and dried with the aim to eliminate the solvent. As a result, the thickness of the obtained membranes was observed to be approximately 100 µm and denoted as sPSU (raw material) and sPSU/x%TiO_2_(B) (composites with different TiO_2_(B) concentrations in weigth).

### 2.5. Characterization and Measurements

#### 2.5.1. H-NMR Analysis

sPSU sulfonation degree was determined by liquid ^1^H-NMR spectroscopy [34]. The spectra of polymers were recorded on a Bruker Avance (Billerica, MA, USA) DPX 300 MHz spectrometer using dimethylsulfoxide (DMSO-d_6_) as a solvent and tetramethylsilane (TMS) as internal reference.

#### 2.5.2. X-ray Diffraction (XRD)

A Philips (Eindhoven, The Netherlands) X’Pert MPD diffractometer was employed for recording the X-ray diffraction patterns, using a CuKα radiation source. This instrument had a (θ/2θ) Bragg–Brentano geometry and it was equipped with a curved graphite monochromator. The measurements were carried out using a 40 kV voltage and 40 mA current. A step scan of 0.02° and a counting time of 0.5 s per step were selected and the data were recorded in the 2θ range between 5 and 60°.

#### 2.5.3. Scanning Electron Microscopy (SEM)

The morphology of TiO_2_(B) nanowires and composite membranes was analyzed using TENEO-FEI (Hillsboro, OR, USA)- field emission scanning electron microscope, FESEM. The images were taken using a STEM 3 detector with Bright Field mode and an acceleration voltage of 30 kV. On the other hand, composite membrane images were obtained with a concentric backscatter detector (CBS) and an energy-dispersive analyzer (EDAX Octane Silicon Drift Detector) employing an acceleration voltage of 2.0 kV. For a better inspection, TiO_2_(B) samples were coated with a very thin conductive layer of Au using a low vacuum coater Leica EM ACE200.

#### 2.5.4. Water Absorption

The water uptake (WU) of the membranes was determined at room temperature by measuring the weight difference between the dry and the swollen samples. The following equation was used to calculate the water uptake values [16]:

(1) $$WU\left(\%\right)=\frac{W_{wet}-W_{dry}}{W_{dry}}\times 100$$

where *W_wet_* and *W_dry_* are the values of the mass of the membrane after and before being immersed in water.

The dry membrane weight (*W_dry_*) was measured after drying the membranes at 60 °C for 24 h. On the other hand, the weight of the wet membranes (*W_wet_*) was determined after immersing the dry samples in distilled water for 72 h at room temperature. The excess water of the surface was quickly wiped off before weighting.

#### 2.5.5. Infrared Spectroscopy (FT-IR)

Fourier transform infrared spectra of the samples were recorded using a FTIR spectrometer Perkin-Elmer (Waltham, MA, USA) Spectrum GX Instrument (resolution of 4 cm^−1^) in the range of 4000–400 cm^−1^. The infrared spectra of TiO_2_(B) nanowires were recorded in KBr pellets. In the case of composites membranes, slices of about 50 µm thickness were used.

#### 2.5.6. Thermogravimetric Analysis

Thermogravimetric analysis (TGA) was employed mainly to study the thermal stability of the polymer membranes. The experiments were carried out in a thermogravimetric analyzer, Perkin Elmer (Waltham, MA, USA) TGA1, in air atmosphere and at a temperature range from 30 to 900 °C (10 °C min^−1^ heating rate) [16]. For all samples, the initial weight was approximately 9 mg.

#### 2.5.7. Mechanical Properties

The equipment used for the thermomechanical characterization of the membranes was a DMA Q800 (TA Instruments, (New Castle, DE, USA). Tensile strength was investigated at 30 °C on membrane samples of constant dimensions (3 mm × 25 mm area and 100 μm thickness). A ramping force from 0.1 to 15 N min^−1^ at 1 Hz was selected and an oscillation amplitude of 15 µm was employed. All measurements were carried out in quintuplicate. Due to the running time of each experiment, between 15–30 min, all the samples were tested in dry conditions.

#### 2.5.8. Impedance Spectroscopy

The ionic conductivity measurements of the obtained membranes were carried out by means of electrochemical impedance spectroscopy technique (EIS), employing a liquid cell with two compartments separated by two O-rings where the membrane was sandwiched [35]. The electrochemical measurements were performed in different HCl solutions (4 × 10^−4^ M < c < 0.1 M) at room temperature. Before testing, the membranes were immersed in HCl (variable concentration) during at least 12 h. The measurements were performed with an impedance/gain-phase analyzer (Solartron (Farnborough, UK) 1260) and an electrochemical interface (Solartron 1287), in a frequency range between 10^−1^ Hz and 1 MHz by using an oscillating voltage of 10 mV. With the aim to study the temperature effects, tests at different temperatures (30–80 °C) were carried out in a Binder KMF 115 (E5.2) constant climate chamber. To ensure reproducibility, a stabilization time of 15 min. was employed before recording the data. After this time, no change in conductivity were observed. The ionic conductivity of the membranes (*σ_m_*) was obtained from the following equation: *σ_m_* = t/RA, where t and A are the thickness and the surface area of the sample, respectively. The resistance (R) is determined from the Nyquist plot by applying the corresponding geometric factors.

#### 2.5.9. MEA Performances

The equipment used for MEA testing was a Scribner 850e multi range fuel cell test system. The measurements were conducted at 100% RH, atmospheric pressure and in a temperature ranged from 50 to 85 °C. As fuel and oxidant compounds, pure hydrogen and pure oxygen gases were employed, respectively, at a flow rate of 200 mL min^−1^. Pt/C catalyst layers (70 wt.% Pt, Paxitech, (Échirolles, France)) (0.5 mg Pt cm^−2^) were used in both anode and cathode. The membrane thickness was around 100 µm.

By using Electrochemical Impedance Spectroscopy, the in situ through-plane proton conductivity of the membranes was determined. The measurements were carried out by using a potentiostat Autolab (Utrecht, The Netherlands) PGStat30 equipped with a FRA module in the temperature range from 50 to 85 °C at 100% RH. Humidified hydrogen (SHE, anode) and nitrogen (cathode) at a flow rate of 200 mL min^−1^ were used. An excitation signal of 10 mV was selected and the measurements were recorded between the frequency range between 10 and 105 Hz. The dc bias potential was 0.45 V.

## 3. Results

### 3.1. Sulfonation of PSU and Preparation of TiO_2_(B) Nanowires

**Figure 1:**
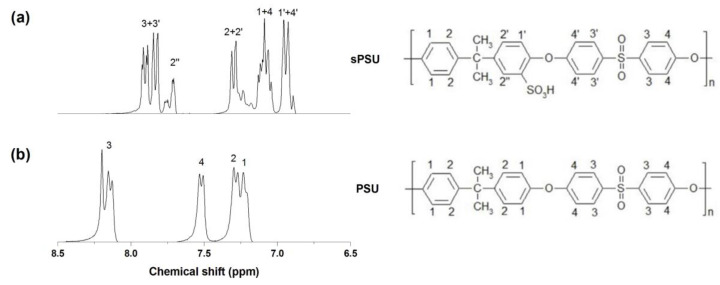
^1^H-NMR spectra of (**a**) sulfonated polysulfone (sPSU) and (**b**) polysulfone (PSU).

The sulfonation degree of PSU was evaluated by ^1^H-NMR spectroscopy. The ^1^H NMR spectra corresponding to PSU and sPSU are shown in Figure 1. The proton resonance at ~7.7 ppm displayed on sPSU spectrum was assigned to the proton adjacent to the new incorporated pendent sulfonic acid. A degree of sulfonation of 0.7 was obtained for sPSU, which was determined by means of a modified formula reported in the literature [36], considering that the polysulfone might be sulfonated up to two sulfonic groups per monomer unit.

### 3.2. X-ray Diffraction

**Figure 2:**
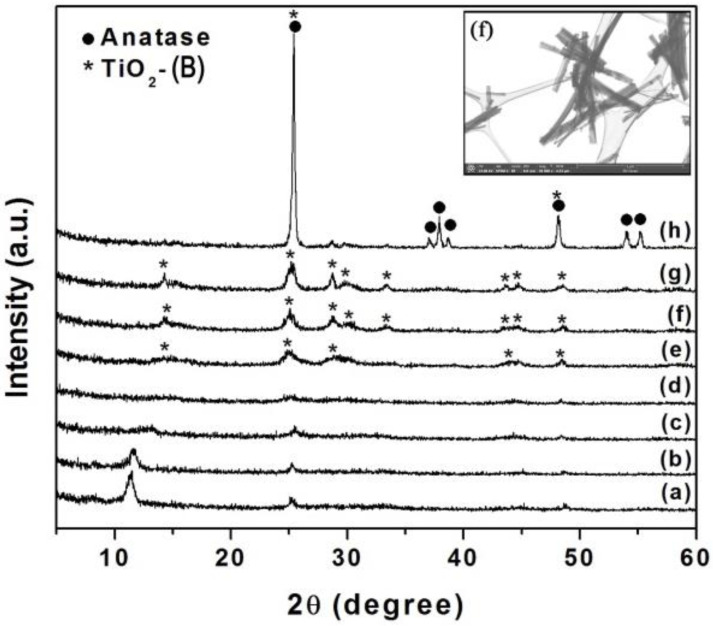
XRD patterns of TiO_2_-related nanowires prepared for 120 h at 150 °C (**a**) as-synthesized, and post-heat treatments calcination for 2 h at (**b**) 100 °C, (**c**) 200 °C, (**d**) 300 °C, (**e**) 400 °C, (**f**) 500 °C, (**g**) 600 °C and (**h**) 700 °C. Peaks corresponded to anatase and TiO_2_(B) are indicated as “•” and “*”, respectively. In inset of figure, a STEM image of TiO_2_(B) nanowires, corresponding to sample (**f**) is displayed.

Figure 2 shows the XRD patterns of the as-synthesized nanowires, which were prepared by the above-mentioned hydrothermal method (at 150 °C for 120 h) (a) and the post-heated samples for 2 h at 100, 200, 300, 400, 500, 600 and 700 °C in (b)–(h). The as-synthesized sample XRD pattern can be assigned to hydrogen titanate (H_2_Ti_3_O_7_) structure, as proposed by Peng’s group [37] and its main peaks can be indexed according to JCPDS 47-0561. Thus, the diffraction peak appeared at 2θ~12° is indexed as (200) reflection and it corresponds to the interlayer spacing of layered titanates. This peak is slightly shifted to higher angles when the sample was heated at both 100 and 200 °C, which has been explained by the dehydration of interlayer water in the titanate [38]. On the other hand, when treating the as-synthesized nanowires at around 400 °C, the transformation from titanate to TiO_2_(B) seemed to proceed and it appeared to be completed between 400 and 600 °C. Therefore, the XRD patterns of samples heated between these temperatures can be identified and indexed as TiO_2_(B) (JCPDS 74-1940). Finally, above 600 °C, the formation of anatase phase began, which is accompanied by an increase of crystallinity. Recent reports of TiO_2_(B) nanowire synthesis showed similar transformation temperature [39]. Thus, TiO_2_(B) nanowires used for the preparation of membranes in the present work were obtained by a heat treatment of titanium oxide nanowires at 500 °C for 2 h (Figure 2f).

**Figure 3:**
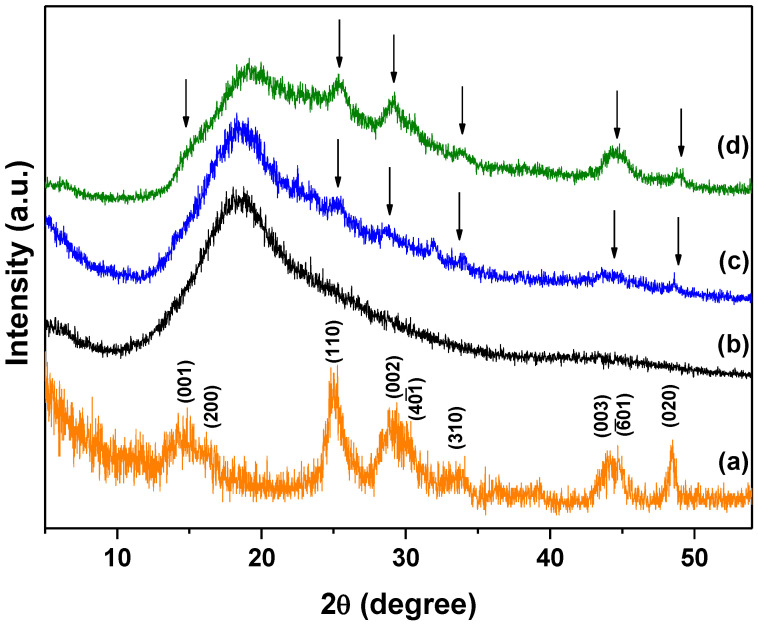
XRD patterns of (**a**) TiO_2_(B) (prepared by heating the as-synthesized titanium oxide at 500 °C for 2 h), (**b**) sPSU membrane, sPSU/2% TiO_2_(B) (**c**) and sPSU/10% TiO_2_(B) (**d**) composite membranes.

XRD patterns of TiO_2_(B) nanowires, sPSU membrane and composite membranes are displayed in Figure 3. sPSU membrane showed a very broad peak at around 18° which reflect the low crystallinity of this material [40]. On the other hand, XRD patterns of TiO_2_(B) nanowires displayed very broad characteristic peaks related with its nano-size scale. Regarding doped membranes, the main reflections of TiO_2_(B) can be distinguished (indicated with arrows in Figure 3). Thus, peaks at 2θ~15°, 25°, 30°, 44° and 48° for the sPSU/10% TiO_2_(B) membranes (Figure 3d) can be seen. The presence of these peaks can also be observed in the sPSU/2% TiO_2_(B) pattern with lower intensity due to the lower concentration of nanowires.

So, the presence of TiO_2_(B) nanowires in the composite membranes have been confirmed by means of X-ray diffraction.

### 3.3. Scanning Electron Microscopy

**Figure 4:**
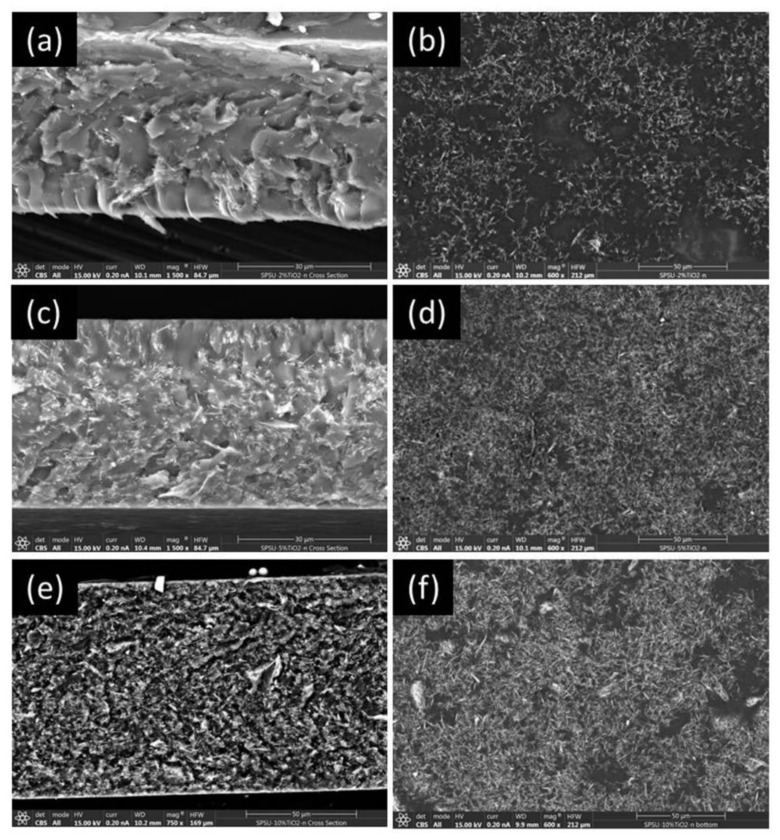
SEM images of the cross-section and surface of the composite membranes with sPSU/2%TiO_2_(B) (**a**,**b**), 5%TiO_2_(B) (**c**,**d**) and 10%TiO_2_(B) (**e**,**f**).

The diameters of the TiO_2_(B) nanowires were between 30 and 50 nm, and the lengths were in the range of 90 to 120 nm (see Figure S1). Furthermore, the elemental analysis of the sample, by means of EDS (Energy Dispersive Spectroscopy), revealed no rest of sodium or chloride on the surface of nanowires coming from the synthesis process. On the other hand, in order to study the dispersion of the inorganic fillers and to confirm their presence in the composite membranes samples, the cross-section and the surface of the prepared membranes were inspected through scanning electron microscopy (Figure 4). The SEM micrographs of the composite membranes show a good distribution of nanowires in the polymer matrix even for sample with low TiO_2_(B) nanowire content (Figure 4a). As increasing the TiO_2_(B) concentration, no agglomeration of nanowires was observed in the polymer matrix even in the case of the sPSU/10% TiO_2_(B) (Figure 4d). The acicular morphology and the lightness of the nanowires probably helps to form good homogenization.

To sum up, SEM micrographs reveal that TiO_2_(B) nanowires show good distribution in the polymer matrix in all TiO_2_(B) concentration range studied (from 2 to 10%).

### 3.4. Water Uptake

Water absorption of membranes is an important feature in PEM due to its intense effects on the dimensional stability, mechanical properties and ionic conductivity of the membranes. Thus, an optimum value of water uptake is necessary to achieve an enough high conductivity, without a detriment of the mechanical properties. The percentages of water uptake obtained for the prepared membranes are shown in Table 1. The results pointed out that the presence of TiO_2_(B) nanowires produced a significant increase of the water absorption from 29.2% for the pristine sPSU to 45.7% and 49.2% for a concentration of 1 and 2 wt.% of TiO_2_(B), respectively. This behavior could be associated with the hydrophilic nature of the TiO_2_ nanowires as mentioned in the introduction, which is related to the presence of hydroxyl groups in their surface [41,42]. This polar surface explains the dipole-dipole interaction between TiO_2_(B) nanowires and water molecules. This fact could be responsible of the significant increase of the water uptake values of polysulfone with the incorporation of just 1 wt.% of TiO_2_(B) nanowires. However, for the sample with the highest TiO_2_(B) nanowire content, the water absorption decreased. This fact can be explained by the high concentration of inorganic nanowires, which reduces the membrane swelling ability as a consequence of its free volume decrease. These results are in agreement with those reported in the study of Slade et al. [43] and Sahin et al. [44], where they observed an increase of the water uptake capacity of TiO_2_ doped Nafion 1100 and PVA membranes. However, Unnikrishnan et al. [22] observed that the water uptake of sPSU membranes was noticed to be decreased with the introduction of TiO_2_ (anatase) content, as the inorganic particles could mask the hydrophilic –SO_3_H groups in the sPSU, leading to a decrease of the value of this property.

To conclude, the water uptake of the membranes is increased until a percentage of around of 70% when the inorganic filler is incorporated into the polymer matrix.

### 3.5. FTIR Analysis

**Figure 5:**
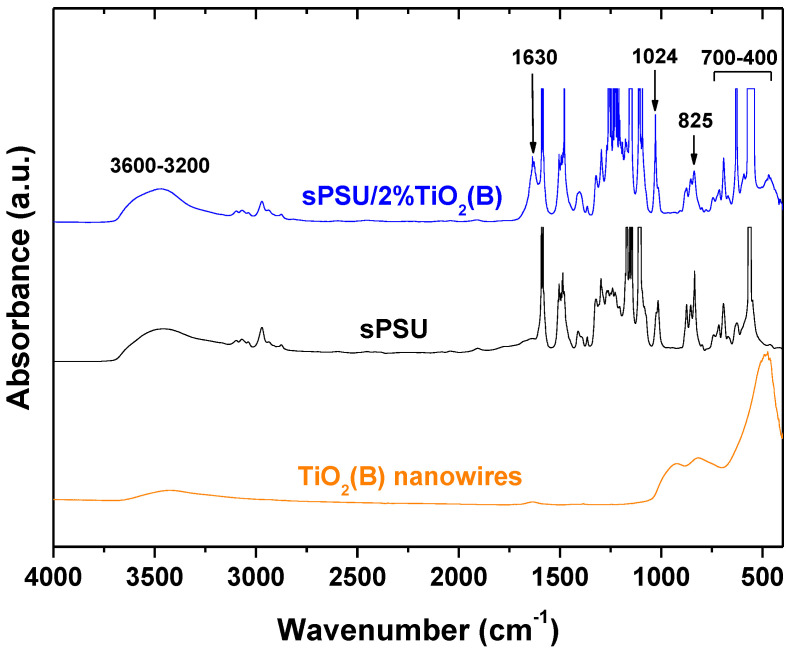
FTIR spectra of TiO_2_(B) nanowires, sPSU and doped sPSU/2%TiO_2_(B) composite membranes. The FTIR peak assignments are collected in Table S1.

The FTIR spectra of sPSU, TiO_2_(B) nanowires and sPSU/2% TiO_2_(B) composite membrane are shown in Figure 5. The spectrum of synthesized TiO_2_(B) nanowires showed an intense peak between 400 and 700 cm^−1^ which is assigned to the Ti–O–Ti stretching vibrations [45] and another peak at around 925 cm^−1^,corresponding to Ti-OH stretching [46]. The broad band between 3200 and 3600 cm^−1^ is identified with the stretching mode of hydroxyl, δ_OH_, while that at 1630 cm^−1^ is correlated to bending modes of -OH groups [47]. Regarding the sPSU membranes, the presence of a characteristic peak at 1024 cm^−1^, assigned to the symmetric stretching of the sulfonate group [48], confirmed that the sulfonation process was successfully carried out. This peak was also present in all studied sulfonated membranes. Also, the Ti^4+^ ions in TiO_2_(B) nanowires can be coordinated by hydroxyl groups [49] leading to an increase in intensity of the formed –OH bands at 3500 cm^−1^ into the polymer matrix. This interaction can be favorable for improving the thermal stability, the optical transparency [50] and the water absorption of the films as observed before. Also, the presence of an intense peak at 1630 cm^−1^ in the doped membrane corresponds to H-O-H bending of the hydrated TiO_2_ which is in agreement with the water adsorption capacity of TiO_2_ nanowires [44,51], justifying the increase of water retention of the composite membranes due to their incorporation into the polymer (see Table 1).

Therefore, the presence of TiO_2_(B) nanowires in the polymer matrix as well as their ability to absorb water molecules can be easily verified by means of FTIR analysis.

### 3.6. Thermal Analysis

**Figure 6:**
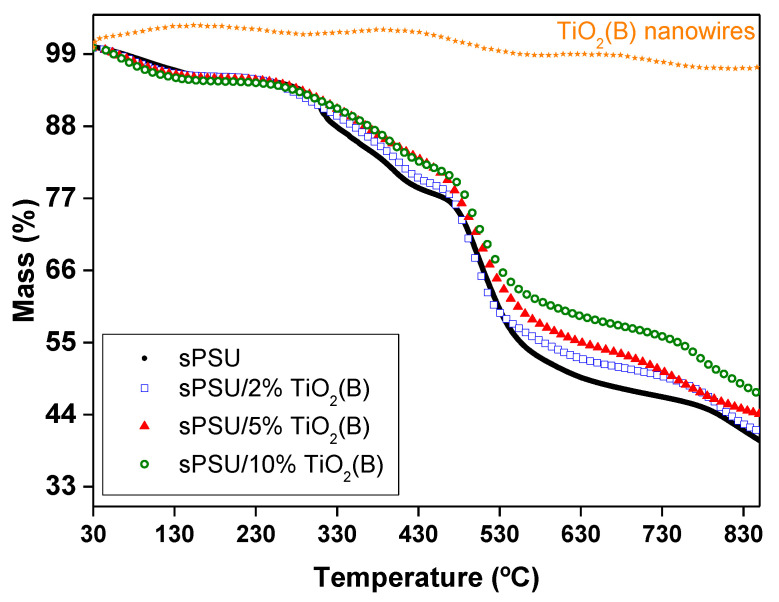
TGA plots of TiO_2_(B) nanowires, sPSU membrane and sPSU/x%TiO_2_(B) composite membranes (x = 2; 5 and 10).

Decomposition curves obtained for TiO_2_(B) nanowires and the different sPSU composite membranes are shown in Figure 6. The thermogravimetric curve of TiO_2_(B) powder showed a high stability of this compound with no remarkable mass change (weight losses less than 3% in the temperature range of 30–850 °C). In the case of the sPSU, three mass losses were observed. The first mass loss identified between 50 and 200 °C is due either to the evaporation of water or desorption of water from the hydrophilic sulfonic groups. After that, between 250 and 450 °C, the desulfonation process occurs, with the corresponding evolution of SO_2_ and SO gases. The last weight loss, which starts around 450 °C, corresponds to the polymer backbone decomposition [12]. Regarding the composite membranes, sPSU/x%TiO_2_(B), although a similar pattern was found, the onset temperature of the last stage was slightly shifted to higher values due to the incorporation of nanowires into the polymer matrix. This shift can be attributed to the interaction between the nanowires and polysulfone which produces a limitation of the movements of polymer chain segments. Thus, the maximum temperature of polymer backbone decomposition was 464, 470, 474 and 482 °C for sPSU and sPSU composites with 2%, 5% and 10% of TiO_2_(B), respectively.

From thermogravimetric analysis, it can be extracted that TiO_2_(B) nanowires slightly delay the thermal decomposition of the composite membranes and exhibit high thermal stability in all cases below 100 °C, that is the operation temperature in this type of fuel cells.

### 3.7. Mechanical Properties

**Figure 7:**
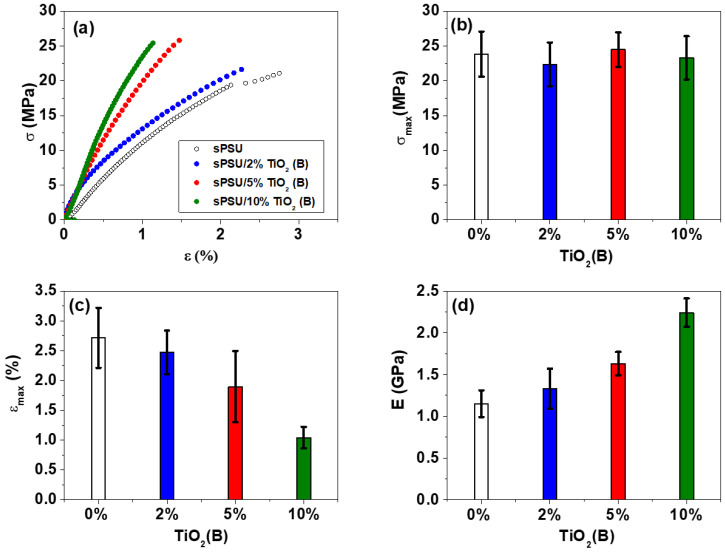
(**a**) Stress (σ)-strain (ε) curves and values of (**b**) maximum stress (σ_max_), (**c**) elongation-to-break (ε), (**d**) Young modulus (E) for sPSU and sPSU/x% TiO_2_(B) membranes (x = 2; 5 and 10).

The mechanical characterization of the membranes must be considered in the design of the fuel cell device as the membranes should present sufficient consistency and mechanical strength, not only to resist the assembly process of the cell but also to withstand operating conditions and to avoid the contact between the electrodes. Figure 7 shows an example of the stress (σ)-strain (ε) curves and the obtained values of the mechanical properties for sPSU and sPSU/x% TiO_2_(B) composites membranes.

The results indicate that the presence of TiO_2_(B) nanowires has little or no effect on the maximum stress. Previous studies reported a decrease in the maximum stress from pristine membranes to those prepared with an inorganic load [52]. On the other hand, Martínez-Morlanes et al. [16] obtained that SiO_2_ doped sPSU exhibited higher strength at break than neat sPSU up to 4 wt.% SiO_2_. However, it can be seen that the value of the elongation at break decreases considerably (by one-third for a concentration of 10 wt.%) with the incorporation of nanowires. These results are similar to that obtained in the study of Shao et al. for the commercial Nafion 115 membrane [53]. This study reported that the neat nafion membranes became brittle due to the presence of different inorganic oxides. It must be highlighted that the low elongation values obtained for sPSU/x%TiO_2_(B) membranes (1–3%) can be explained due to their lack of hydration, as in all the cases the membranes were measured under dry conditions. Therefore, properly humidified membranes are expected to reach much higher elongation.

As a consequence of the loss of elongation observed due to the presence of TiO_2_(B) nanowires, the material is stiffened, and this is also observed in an increasing on the Young’s modulus, which was estimated from the slope of the stress–strain plot at small strains. Thus, sPSU/x%TiO_2_(B) composites showed an almost 95% increase in Young’s modulus due to the addition of 10 wt.% TiO_2_(B) nanowires. The average values of this property were 1.15 and 2.24 GPa for sPSU and sPSU/10% TiO_2_(B), respectively. The interaction of the filler with the polymer matrix makes the polymer lose flexibility, which is due to the brittle nature of the inorganic filler as observed in earlier studies [54].

To conclude, the stress-strain curves of the membranes here studied reveal that TiO_2_(B) nanowires in the polymer membrane have a small effect on the maximum stress, increase the Young’s modulus and decrease the elongation at break, making the composite membrane less flexible.

### 3.8. Ionic Conductivity

**Figure 8:**
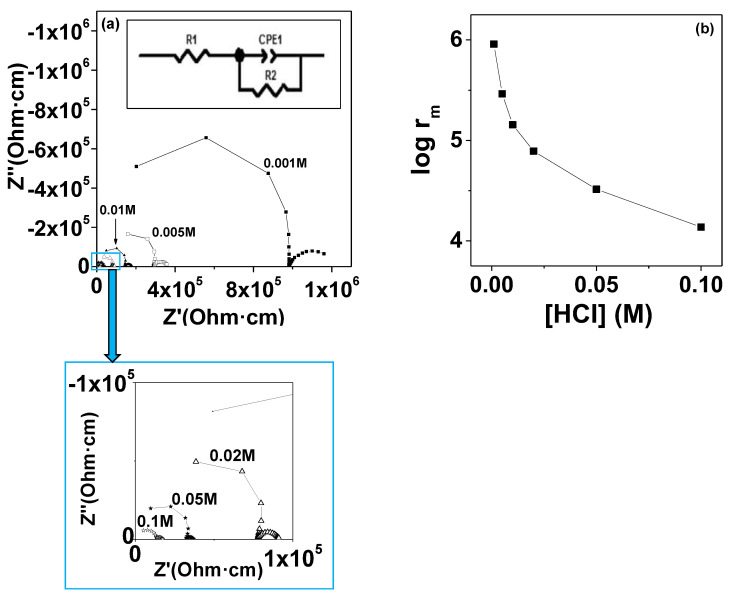
(**a**) Complex impedance plots of sPSU/10% TiO_2_(B) composite at different HCl concentrations. In the inset, the equivalent circuit used for the data fitting is also displayed and (**b**) the ionic resistivity evolution for different HCl solutions.

The proton conductivity of the prepared membranes was measured by EIS immersing the membranes in aqueous solution of HCl with different concentrations. As an example, Figure 8a shows the impedance plot of sPSU/10% TiO_2_(B) composite as a function of different HCl concentrations.

**Figure 9:**
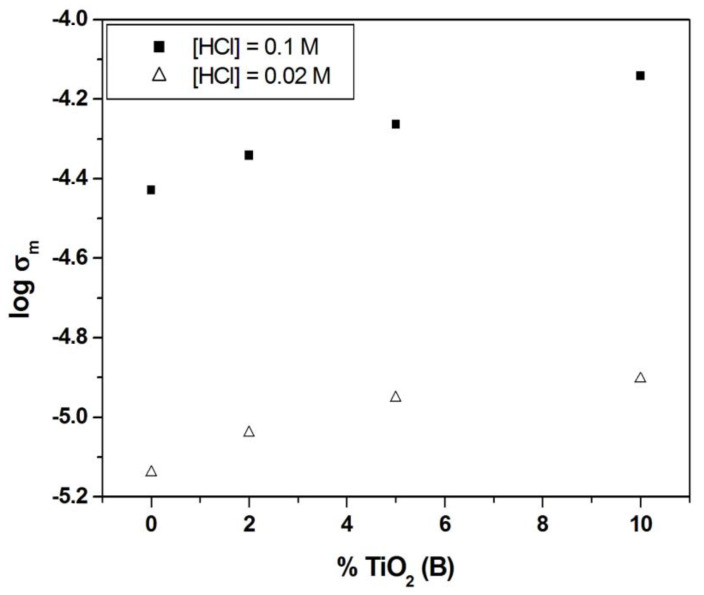
Ionic conductivity of sPSU and sPSU/x%TiO_2_(B) composite membranes (x = 2; 5 and 10) measured at room temperature ([HCl] = 0.02 M and [HCl] = 0.1 M).

For all the samples, two semicircles are distinguished. The first semicircle, at high frequency, was related to the membrane capacitance while the second arc, clearly deformed and located at low frequency, is correlated to the diffusion of electroactive species. Also, the fact that the high frequency arc did not pass through the origin of the plot indicated the presence of a resistive contribution which could be related to the electrolyte solution. As expected, the resistivity associated to all composite membranes (obtained from the intersection of the high frequency semicircle (left) with the real axis in Nyquist plot) decreased with the concentration of HCl (Figure 8b). It showed a possible dependence of this parameter with the electrolyte solution, which is embedded in the membrane matrix [32,50]. Regarding to the composites, the conductivity at room temperature obtained for all sPSU/x%TiO_2_(B) membranes is shown in Figure 9.

It can be seen from Figure 9 that the presence of TiO_2_(B) nanowires slightly improved the ionic conductivity of sPSU/x%TiO_2_(B) composite membranes in all the TiO_2_(B) concentration range studied. This result was attributed to the hydrophilic nature of titanium oxide nanowires and the ability of this compound to increase the water uptake of the sPSU as observed previously.

Thus, for the highest HCl concentration ([HCl] = 10^−1^ M), the ionic conductivity of sPSU showed a 94% increase due to the addition of 10% TiO_2_(B) nanowires, reaching a value around 7 × 10^−5^ S·cm^−1^. The continuous increase of the conductivity with the increases of TiO_2_(B) nanowires indicated a high homogeneity of the filler through the membrane, even for the highest concentration of reinforcement.

**Figure 10:**
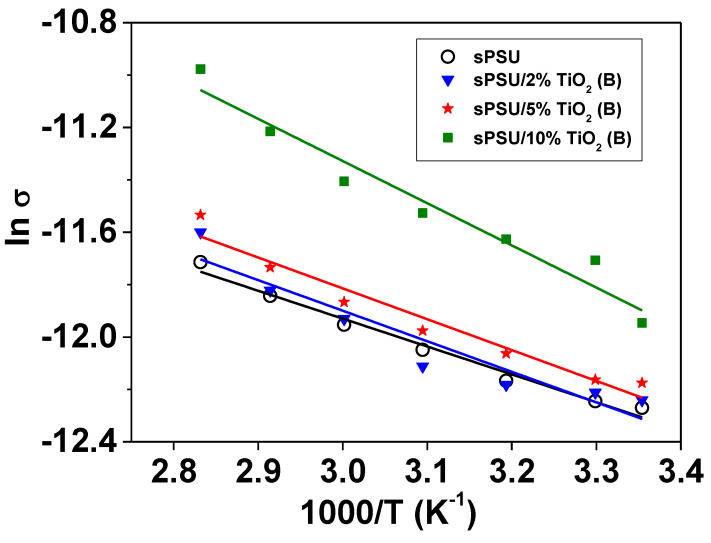
Evolution of proton conductivity with temperature for to the different sPSU/x%TiO_2_(B) composite membranes (x = 2; 5 and 10).

On the other hand, the ionic conductivity variation with temperature of the studied membranes is displayed in Figure 10. Thus, the proton conductivity values of sPSU membranes containing 2 and 5 wt.%TiO_2_(B) nanowires were close to that of neat sPSU membranes. However, when the concentration of reinforcement was increased to 10 wt.%, the conductivity of the composites was higher in comparison with sPSU in all temperature range. This increase of the conductivity shown by the composites is the result of the higher thermal stability due to the presence of TiO_2_(B) nanowires. These results agree with those obtained by Devrim et al. [17] as they observed that the proton conductivity of sPSU/titanium oxide composite membranes increased with temperature.

Finally, the activation energies of all doped membranes were obtained by linear fitting of Figure 10 data. The obtained values were 8.8, 9.7, 9.8 and 13.4 kJ mol^−1^ corresponding to sPSU, sPSU/2%TiO_2_(B), sPSU/5%TiO_2_(B) and sPSU/10%TiO_2_(B), respectively. The results showed a slight increase of this parameter with the amount of TiO_2_(B) nanowires. However, the obtained low values indicated that the predominant mechanism of proton transport in the neat polymer and composite membranes was the vehicular mechanism [55]. Similar behavior had been found in other sPSU composite membranes [16], indicating that transport properties are mainly governed by the polymer nature.

In the light of these results, proton conductivity of the composite membranes does not vary significantly when TiO_2_(B) nanowires are incorporated into the polysulfone matrix although a clear tendency was observed; the ionic conductivity improves with increase in the percentage of the inorganic filler. In addition, composite membranes show higher ionic conductivity than sPSU membranes at elevated temperatures (near to 80 *°*C) and this fact is attributed to the effect of TiO_2_(B) nanowires which are able to retain water molecules under these experimental conditions.

### 3.9. Fuel Cell Test

**Figure 11:**
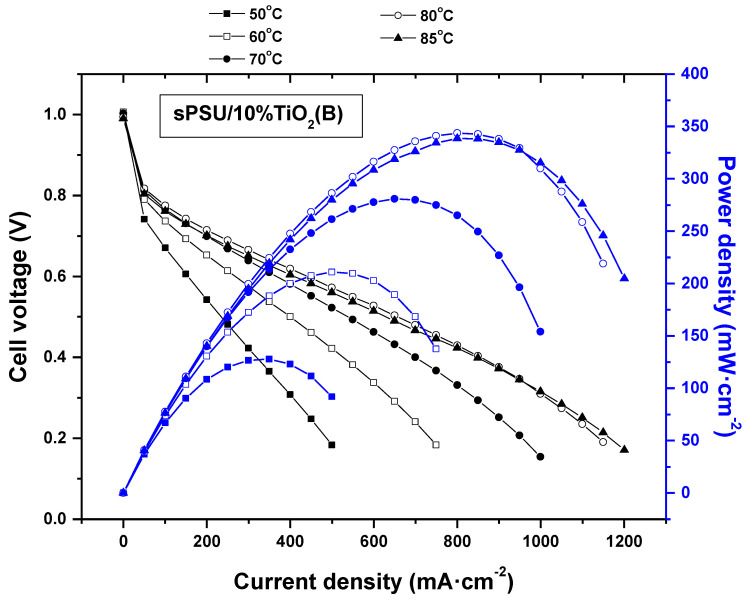
Polarization and power density curves of sPSU/10%TiO_2_(B) composite membranes registered in the 50–85 °C temperature range.

To evaluate the performance of the composite membranes on single cells, the membrane with the highest ionic conductivity, sPSU/10%TiO_2_(B), was selected to be tested. Figure 11 shows the polarization and power density curves of the sPSU/10%TiO_2_(B) membrane. Measurements were conducted on 5 cm^2^ active area single cells, at atmospheric pressure of reactant gases (H_2_/O_2_), different cell temperatures, and fully hydrated conditions (100% RH).

The performance of sPSU/10%TiO_2_(B) membrane improved with increase in temperature reaching to a maximum current density of 1200 mA·cm^−2^ and a maximum power density of 340 mW·cm^−2^ at 85 °C (vs. 500 mA·cm^−2^ and 130 mW·cm^−2^ at 50 °C). These values are of the same order of magnitude as those obtained for Nafion 117 which performs 310 mW cm^−2^ and around 999 mA cm^−2^ power and current densities, respectively, at 70 °C and 100% RH [56]. When comparing with similar systems, an improvement in the performance of these composite membranes is observed. Thus, Devrim et al. [21] prepared composite membranes based on sPSU and TiO_2_, measuring a maximum power of 240 mW cm^−2^ at 85 °C.

By EIS, the in situ through-plane proton conductivity on the MEA of sPSU/10%TiO_2_(B) membrane (σ_m_ MEA) was also determined. Measurements were performed at atmospheric pressure and 100%RH with humidified gases. At high temperature, the proton conductivity of pristine sPSU membrane decreases from 23.4 mS cm^−1^ at 80 °C to 23.2 mS cm^−1^ at 85 °C, whereas in the case of sPSU/10%TiO_2_(B) membrane, there is a slight increase (from 20.0 mS cm^−1^ at 80 °C to 20.10 mS cm^−1^ at 85 °C). This behavior can be associated with the higher water retention of the hybrid membrane (WU% = 29 ± 3), compared with the pure membrane (WU% = 41.1 ± 5.1) which would improve the ionic conductivity. Similar results were observed in Nafion/TiO_2_ nanowire composite membranes at elevated temperatures [57]. In the same line, Devrim et al. [21] observed that the addition of TiO_2_ powder to sPSU reduces the ionic resistances at high temperatures.

To sum up, the behavior of sPSU/10%TiO_2_(B) membrane in the fuel cell is similar to that of Nafion 117 considering this as a reference material. In addition, the ionic conductivity determined by both in situ and ex situ measurements follow a similar behavior at high temperature, revealing thus the great influence of TiO_2_(B) nanowires on the electrochemical properties of the composite membranes.

## 4. Conclusions

In this work, polymer electrolytes for fuel cells based on sPSU/TiO_2_(B) nanowire composites were obtained via solvent casting. The preparation of TiO_2_(B) nanowires was successfully carried out by hydrothermal method, followed by a heating treatment at 500 °C for 2 h, as confirmed by XRD and FESEM experiments. Also, these techniques together with FTIR spectroscopy were used to confirm the homogeneous distribution TiO_2_(B) nanowires in the polymer matrix, which is probably due to the acicular morphology and the lightness of the nanowires. The TGA results indicate that these hybrid membranes have a good thermal resistance which is slightly improved with the consecutive addition of nanowires. Furthermore, the water adsorption capability of TiO_2_(B) nanofiller improved the ability of the composites to absorb water, due to the hydrophilic nature of the membranes. The composite sPSU/x%TiO_2_(B) membranes reached between 40 and 50% of water absorption. On the other hand, a negligible variation of the maximum stress with the addition of TiO_2_(B) nanowires was observed. However, the presence of inorganic nanowires produced a decrease of elasticity, decreasing the % elongation at break of the composite membranes from 3 (sPSU) to 1% (sPSU/10%TiO_2_(B)). Regarding to proton conductivity, the impedance spectroscopy study showed that the ionic conductivity of sPSU at room temperature was enhanced in all TiO_2_(B) concentration range up to 7.1 × 10^−5^ S cm^−1^ for sPSU/10%TiO_2_(B) ([HCl] = 0.1). Also, at higher temperatures, the conductivity of the sPSU/x%TiO_2_(B) composites (x = 2, 5 and 10) were higher in comparison with neat polymer. This increase can be attributed to both, the higher water uptake and the increase of the thermal stability due to the presence of the nanowires. The maximum power density obtained for the most conducting membrane, i.e., sPSU/10%TiO_2_(B) membrane, was 340 mWcm^−2^ at 85 °C. These results revealed the acceptable behavior of these materials, especially when these data are compared to the commercial Nafion 117, which make these proton exchange membranes a promising candidate for their use in PEMFCs.

To sum up, in comparison to the neat polymer, sPSU/TiO_2_(B) nanowire composites show better thermal stability, higher ability to absorb water and improved ionic conductivity at high temperature. It is true that no significant variations were observed, due probably to the small amount of TiO_2_(B) nanowires dispersed in the polymer matrix. So, in view of these results, as a continuation of the work presented in this paper, the synthesis of new membranes with a large amount of titanium oxide and less quantity of polymer, i.e., the minimum quantity necessary to guarantee the consistency of the material, could be promising. These materials would have the advantages inherent to the TiO_2_(B) nanowires with improved mechanical stability associated to the presence of sPSU.

## Figures and Tables

**Table 1:** Water absorption percentage of sPSU membrane and sPSU/x%TiO_2_(B) composite membranes.

Membrane	Water Uptake (%)
sPSU	29 ± 3
sPSU/1%TiO_2_(B)	45 ± 3
sPSU/2%TiO_2_(B)	49 ± 3
sPSU/5%TiO_2_(B)	49 ± 4
sPSU/10%TiO_2_(B)	41 ± 5

## Data Availability

No applicable.

## Supplementary Materials

The following are available online at https://www.mdpi.com/article/10.3390/polym13122030/s1, Figure S1: STEM image of TiO_2_(B) nanowires synthesized by the hydrothermal method and employed for sPSU/x%TiO_2_(B) membranes, Table S1: FTIR peak assignment for sPSU/2%TiO_2_(B) composite membrane.
