# Supplementary material for: Sulfonated Polysulfone/TiO2(B) Nanowires Composite Membranes as Polymer Electrolytes in Fuel Cells
Source: Polymers (Basel). 2021 Jun 21;13(12):2030. doi: 10.3390/polym13122030 (PMC8234381; doi:10.3390/polym13122030)
Supplement: Supplementary file 1 [file polymers-13-02030-s001.zip › polymers-1250654-supplementary.pdf]

Supplementary material

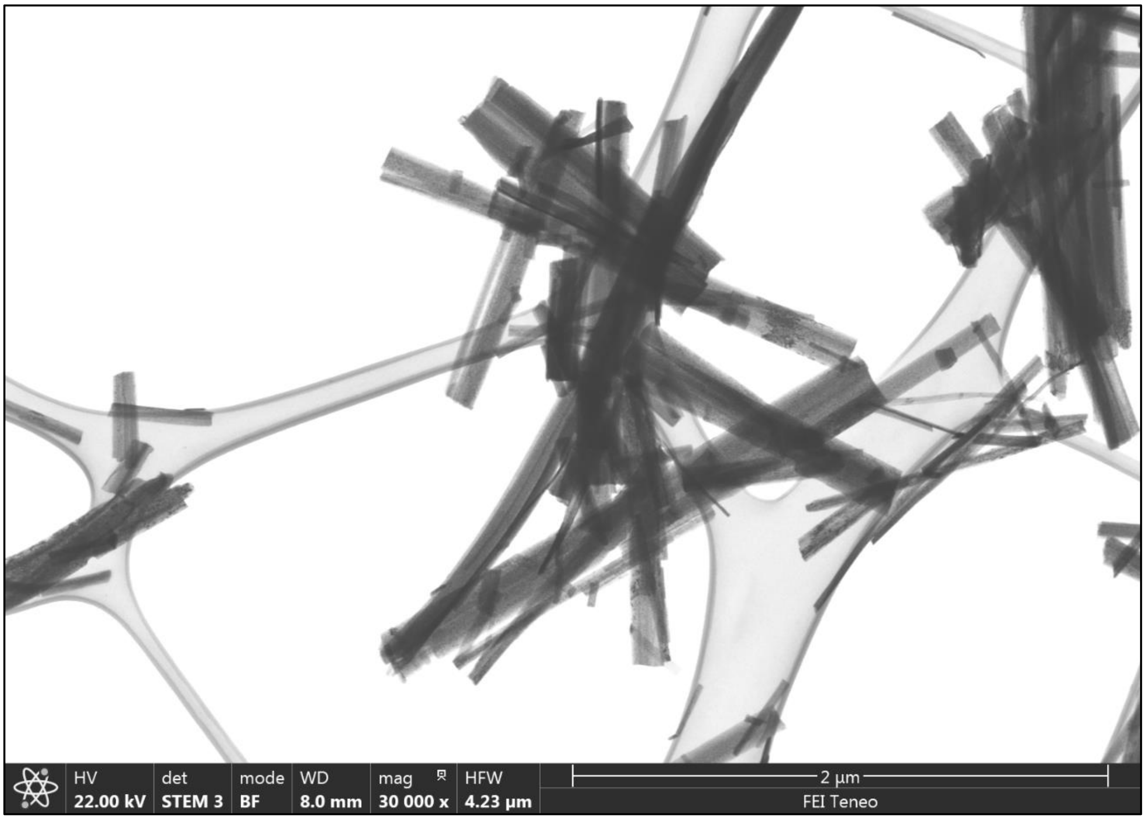

Figure S1. STEM image of TiO_2 (B) nanowires synthesized by the hydrothermal method and employed for sPSU / x% TiO_2 (B) membranes.

Table S1. FTIR peak assignment for sPSU/2%TiO_2(B) composite membrane.

**Wave number (cm^{-1})**	**Vibration type**	**Assignment**
400-700	Stretching	Ti-O-Ti
925	Stretching	Ti-OH
1024	Stretching	$-SO_3H$
1630	Bending	-OH
3200-3600	Stretching	δOH
